# A one-dimensional high-order commensurate phase of tilted molecules

**DOI:** 10.1039/d2cp00437b

**Published:** 2022-03-30

**Authors:** Anthony Thomas, Thomas Leoni, Olivier Siri, Conrad Becker, Martin Unzog, Christian Kern, Peter Puschnig, Peter Zeppenfeld

**Affiliations:** Aix Marseille University, CNRS, CINaM UMR 7325, Campus de Luminy 13288 Marseille cedex 09 France peter.zeppenfeld@jku.at; Institute of Physics, University of Graz, NAWI-Graz, Universitätsplatz 5 8010 Graz Austria; Institute of Experimental Physics, Johannes Kepler University Linz, Altenberger Straße 69 A-4040 Linz Austria

## Abstract

We report on the formation of a high-order commensurate (HOC) structure of 5,14-dihydro-5,7,12,14-tetraazapentacene (DHTAP) molecules on the highly corrugated Cu(110)–(2 × 1)O surface. Scanning tunnelling microscopy shows that the DHTAP molecules form a periodic uniaxial arrangement in which groups of seven molecules are distributed over exactly nine substrate lattice spacings along the [1̄10] direction. DFT-calculations reveal that this peculiar arrangement is associated with different tilting of the seven DHTAP molecules within the quasi one-dimensional HOC unit cell. The orientational degree of freedom thus adds a new parameter, which can efficiently stabilize complex molecular structures on corrugated surfaces.

## Abstract 2

We report on the formation of a quasi-1D high-order commensurate (HOC) structure of dihydro-tetraazapentacene (DHTAP) molecules on a Cu(110)–(2 × 1)O surface and its stabilization through internal degrees of freedoms, namely the molecular tilt.

## Graphical abstract

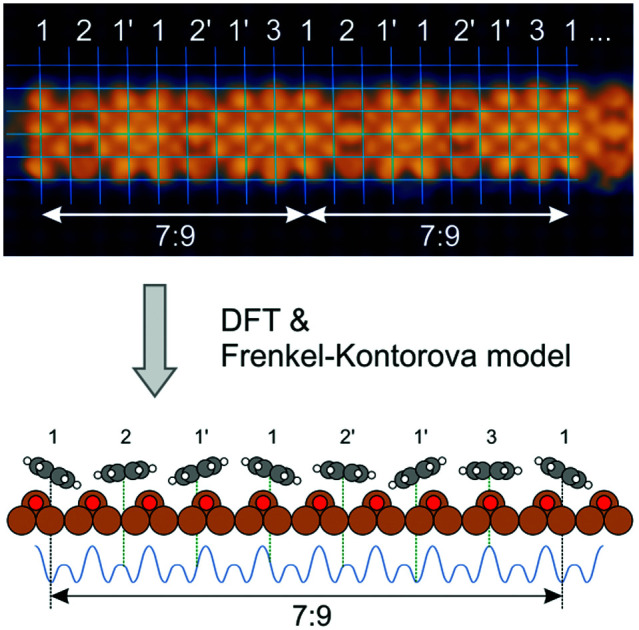

Atoms or molecules adsorbed on crystalline substrates often form regular patterns in a well-defined epitaxial relationship with the substrate surface. Such superstructures are the result of the intricate interplay between adsorbate-substrate and adsorbate–adsorbate interactions.^1^ A strong lateral corrugation of the substrate-adsorbate potential often results in commensurate structures, where the adsorbates are located on identical, energetically preferred adsorption sites in complete registry with the substrate. On the other hand, the lateral interactions between neighbouring adsorbates tend to favour structures in which the mutual distance between the adsorbates is close to their natural one, which is generally incommensurate with the substrate lattice. Besides the fundamental commensurate (C) and fully incommensurate (IC) structures more complex structural coincidences such as domain wall phases, rotational epitaxy and moiré structures can occur, depending on the relative size of the competing interactions and the natural lattice misfit.^2,3^ The relative stability of these different phases also depends on external parameters like the surface coverage and temperature, which can give rise to complex phase diagrams with a variety of solid phases with different superstructures.^4,5^ The simplest case is that of a (quasi) one-dimensional (1D) arrangement of atoms or molecules with natural spacing *b* on a corrugated surface with periodicity *a*, corresponding to a nominal lattice mismatch *δ* = (*b*—*a*)/*a*. Such systems can be treated analytically within the Frenkel–Kontorova model, which assumes a sinusoidal substrate corrugation potential *V*(*x*) = *V*_0_sin(2π*x*/*a*) and a harmonic coupling (with force constant *k*) between neighbouring adsorbates.^6^ The energetically stable solutions to this model depend on the three parameters *δ*, *V*_0_ and *k* and include fundamental C, IC and domain wall phases as well as high-order commensurate (HOC) phases. The latter are characterized by a commensurate coincidence lattice, in which *m* > 1 adsorbates cover a commensurate distance *n* × *a* of the substrate lattice. HOC phases tend to minimize the average lattice mismatch of the coincidence unit cell, *i.e.*, *m* × *b* ≈ *n* × *a*. However, an (*m*: *n*) HOC commensurate phase in which the *m* adsorbate particles are equally spaced within the *n* × *a* HOC unit cell is energetically not preferred against an incommensurate phase in the case of a purely sinusoidal substrate corrugation potential.^2^ Instead, a HOC phase gains its stability by either (1) local lateral relaxations of the adsorbates within the HOC unit cell towards the nearest minima of the corrugation potential, (2) a finite domain size, where the adsorbates at the border are located in energetically preferred adsorption sites, or (3) a deviation of the corrugation potential from a simple sinusoidal shape.

In fact, quasi 1D structures can be realized experimentally using anisotropic substrates such as fcc(110) surfaces. For instance, a combined experimental and theoretical study has demonstrated the existence and the range of stability of various uniaxial (*m*: *n*) HOC phases of Xe on Cu(110) as well as the transition between these HOC phases as a function of coverage and temperature.^7^ While there is plenty of evidence for HOC structures in simple atomic adsorbate systems, there are only few documented examples of (2D) HOC structures of inorganic or organic molecules.^8–10^ Yet the structures of molecular species are particularly interesting since molecules provide additional degrees of freedom, such as their relative orientation and conformational flexibility, which can potentially contribute to the stabilization of HOC phases.

Here we report on a novel molecular model system, namely 5,14-dihydro-5,7,12,14tetraazapentacene (DHTAP)^11,12^ adsorbed on the Cu(110)–(2 × 1)O surface. Scanning tunnelling microscopy (STM) in combination with density functional theory (DFT) calculations reveal that the DHTAP molecules form a (7 : 9) quasi-1D HOC phase stabilized by the different tilting of the *m* = 7 molecules arranged over *n* = 9 substrate lattice spacings.

The experiments were carried out in an ultrahigh vacuum (UHV) system with a base pressure below 10^−10^ mbar. The Cu(110) single crystal was cleaned by repeated cycles of Ar^+^ ion sputtering and thermal annealing at 750 K until a clean and well-ordered surface was observed in scanning tunnelling microscopy. The clean Cu(110) surface was then exposed to 30 L of oxygen at 570 K, resulting in the well-known Cu(110)–O(2 × 1) reconstruction. The DHTAP molecules were thermally evaporated from a quartz crucible with a deposition rate of about 1 ML per hour††Here we define the monolayer as the dense packing of DHTAP molecules arranged into a (9 × 9) coincidence lattice with 2 × 7 = 14 molecules per unit cell, see Fig. 2(b). on a freshly prepared surface kept at room temperature. The STM experiments were performed under UHV conditions with a commercial low-temperature STM (Omicron) using electrochemically etched W-tips. The LT-STM was operated at liquid nitrogen temperature (78 K for both sample and tip) in constant current mode. Fig. 1(a) shows an STM image after deposition of about 0.5 ML of DHTAP on the Cu(110)–(2 × 1)O surface. The molecules adsorb with their long axis parallel to the [001] direction and aggregate into linear chains oriented in the [1̄10] direction. Compared with the atomically resolved substrate lattice (highlighted by grid lines in Fig. 1(a)), we find that the molecules are imaged with different contrast and shape depending on their relative position with respect to the Cu–O rows: molecules with their axis near the top of a Cu–O row (3) or between two rows (2, 2′) appear straight, whereas molecules with a certain offset from the Cu–O rows (1, 1′) have a curved “croissant-like” contour with the middle part closer to the nearest Cu–O row than the extremities. Moreover, the contrast changes in a periodic fashion such that groups of 7 molecules exactly span 9 Cu–O row spacings. This coincidence pattern can be clearly assigned to a quasi-1D high-order commensurate (7 : 9) superstructure. At this coverage, the molecular chains also start to locally assemble into 2D islands (Fig. 1(b)) with neighbouring chains separated by 4.5 Cu lattice spacings (4.5 × 0.361 nm = 1.62 nm). These islands thus exhibit a (2 : 9) coincidence with two chains of DHTAP molecules spanning 9 lattice spacings in the [001] direction.† This also explains the alternating imaging contrast of neighbouring chains (see also Fig. 3(a)). Yet, the 1D periodicity and the apparent shapes of the molecules within each individual chain is not affected by the 2D island formation and there is no well-defined spatial correlation between the phase of the 1D patterns across neighbouring chains. This indicates that the ordering of the DHTAP molecules is dominated by the corrugation and molecular interactions along the [1̄10] direction. Therefore, the adsorption of DHTAP on the Cu(110)–(2 × 1)O surface can be viewed as a model system of a one-dimensional (1D) molecular high-order commensurate structure. The reduction of the effective dimensionality from 2D to 1D allows us to cope with the added complexity associated with the additional degrees of freedom of the flexible molecules within the framework of rigorous (*ab initio*) calculations.

**Fig. 1:**
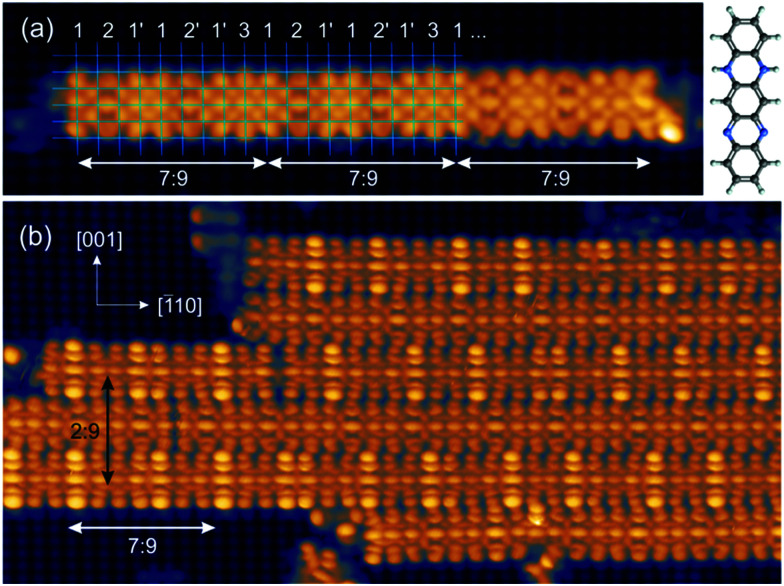
STM images of the Cu(110)–O(2 × 1) surface covered with 0.5 ML of DHTAP. Tunnelling current: *I* = 10 pA (a), *I* = 20 pA (b), sample bias: *V*_S_ = +0.5 V (a), *V*_S_ = −0.5 V (b). The white and black arrows indicate the HOC coincidences (7 : 9) and (2 : 9) along the [1̄10] and [001] direction, respectively. The numbers in (a) refer to different adsorption sites and apparent shapes of the molecules (1, 2, 3) and their mirror images (1′, 2′). A ball-and-stick model of a single DHTAP molecule is shown in the upper right corner.

For theoretically determining the adsorption geometry, we employ the repeated slab approach with a thickness of 5 Cu layers, a vacuum layer of 15 Å and lateral dimensions of 10.2 Å × 21.6 Å utilizing the VASP code.^13,14^ Using a van der Waals corrected^15^ generalized gradient approximation (GGA) based on the PBE functional,^16^ we have obtained the most favourable adsorption geometry with a binding energy of 2.06 eV when the molecule is situated between the Cu–O rows and exhibits an azimuthal tilt of about 25° (configuration A in Fig. 2(a)). To rationalize the different adsorption sites of the DHTAP molecules within the HOC unit cell, we have mapped out the entire potential energy landscape *V*(*x*, *ϕ*) for a single DHTAP molecule by varying its lateral position (*x* ∈ [0, 2*a*], *a* = 2.55 Å) within the (2 × 1)O unit cell and the azimuthal tilt angle *ϕ* (in steps of 5°). Note that for each fixed value of *x* and *ϕ* the adsorption height *z* was optimized in the calculation. The result presented in Fig. 2(b) clearly illustrates the relevance of the molecular tilt on the adsorption energy. In particular, there is a strong correlation between *x* and *ϕ,* which results in the minimum potential energy path (white line in Fig. 2(b)) and gives rise to an effective corrugation potential *V*(*x*) plotted as a function of the lateral position *x* in Fig. 2(c). The variable tilt of the molecule thus leads to a peculiar double-well structure with two mirror symmetric potential minima in which the molecules are adsorbed in a tilted configuration (A, A′) with a lateral offset *x* of about 1.5 Å either to the right or left of a Cu–O row. In between (B) and above (C) the Cu–O rows the molecules lie flat on the surface and are less strongly bound by 0.17 and 0.36 eV, respectively. Besides the tilt angle *ϕ* also the adsorption height varies by about 0.3 Å with the lateral position *x*.

**Fig. 2:**
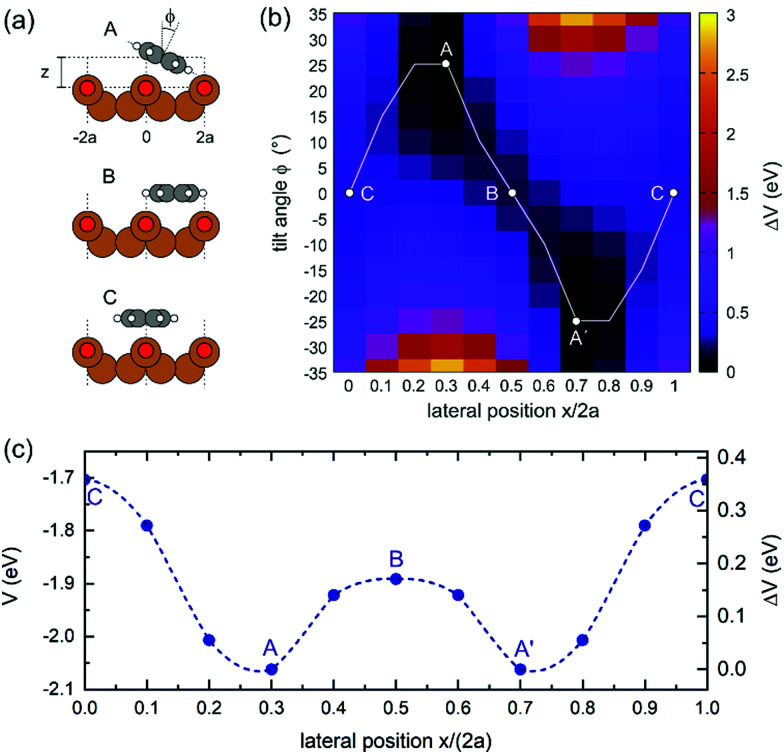
(a) Schematic of the adsorption configurations for three selected positions (A, B, C) of a single DHTAP molecule on the Cu(110)–(2 × 1)O surface. (b) Binding energy as a function of the lateral position *x* ∈ [0, 2*a*] along the [1̄10] direction and the azimuthal tilt angle *ϕ*. The minimum energy path is indicated by the white line and yields the effective 1D corrugation potential shown in (c). Filled symbols in (c) represent the calculated values, the dashed line is a periodic spline interpolation.

Yet, according to the DFT results, the molecules are not bent or distorted, such that the azimuthal tilt angle *ϕ* is the only but central additional parameter that affects the minimum adsorption energy. For a direct comparison of the theoretical findings with the experimental data, STM images were simulated for the most prominent configurations of the DHTAP molecule on the Cu(110)–(2 × 1)O surface, namely the energetically most favourable site (A), where the molecule is in the tilted position, a molecule centred between two Cu–O rows (B) and the adsorption site right above a Cu–O row (C). Using the Tersoff–Hamann approximation,^17^ which assumes tunnelling from/into an atomic s-orbital at the tip, we have evaluated the local density of states in energy windows of ±0.5 eV with respect to the Fermi level. The results for negative energies (occupied states) are compared in Fig. 3 with the experimental STM images recorded for the same sample bias *V*_S_ = 0.5 V. There is a good agreement of the apparent shapes of the measured (2, 1, 3) and the simulated STM images (B, A, C), respectively, whose nodal structure essentially resembles the shape of the highest occupied molecular orbital of DHTAP.^12^ This allows an unequivocal identification of the adsorption sites and tilt of the molecules.‡‡Note that the experimental STM images appear rather symmetric while the calculated ones show a clear difference between the NH and the N positions of the DHTAP molecule. This is because the STM calculations were performed for individual molecules. Indeed, DFT calculations for pairs of DHTAP molecules reveal that two parallel molecules arrange in a head-to-tail fashion, where the NH and N groups of neighbouring molecules form N–H–N bonds at both ends of the molecules, thus yielding a more symmetric electron density distribution. In particular, the croissant-like shape of the species of type (1) in Fig. 1 can now be interpreted to arise from a significant tilt of the DHTAP molecule towards the nearest Cu–O row and the asymmetric bonding configuration of the molecules, rather than to an actual bending or deformation of the molecule. Moreover, the DFT calculations (Fig. 2) reveal that the molecules of type (1) and the mirror symmetric configuration (1′) exhibit the largest tilt and are closest to the energetically most preferred adsorption sites A or A′, respectively. This is also consistent with the experimental observation, that chains of DHTAP molecules in the STM images (see Fig. 1) almost always start or end with a croissant-shaped molecule, *i.e.*, a tilted DHTAP molecule of type (1) or (1′). In fact, a termination with such energetically preferred molecules stabilizes any HOC phase with finite extent.^2,3^ DHTAP molecules of type 2 and 2′ lie between two Cu–O rows (configuration B) but appear slightly shifted to the left or right of the centre position, respectively (see Fig. 1). Finally, the molecules of type 3 are located near the top of a Cu–O row, which corresponds to the least favourable adsorption configuration C. Combining the experimental data and the DFT results we can now derive a tentative model of the arrangement of the 7 DHTAP molecules within the HOC unit cell comprising 9 substrate unit cells. If we assume regularly spaced, non-interacting DHTAP molecules, *i.e.*, a fixed nearest neighbour distance *b* = 9/7 × (2*a*), we can determine the intrinsic stabilization of a HOC phase by calculating the sum of the binding energies 
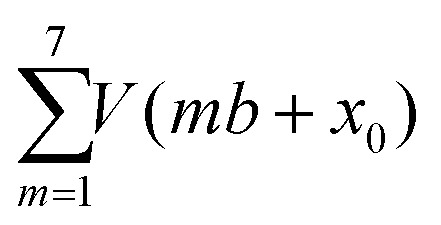
 as a function of the lateral shift *x*_0_ across the substrate unit cell. For the corrugation potential *V*(*x*) we can either use a linear interpolation of the DFT results or the periodic spline curve drawn as a dashed line in Fig. 2(c). In either case we find two stable solutions, one for *x*_0_ = 0 (where one DHTAP molecule rests on top of a Cu–O row) the other for *x*_0_ = *a* (with a DHTAP molecule centred right between two Cu–O rows). The stabilization energy is only of the order of 10 meV per unit cell with a slight preference (by 5 meV) for *x*_0_ = *a*. The two structures are depicted in Fig. 4. From the observed registry with the substrate lattice (see Fig. 1), we conclude that the HOC superstructure is well reproduced by the model in Fig. 4(b) with *x*_0_ = *a*. Besides the intrinsic stabilization, a slight relaxation of the molecules along the negative gradient of the corrugation potential could substantially increase the stability of the HOC phase.

**Fig. 3:**
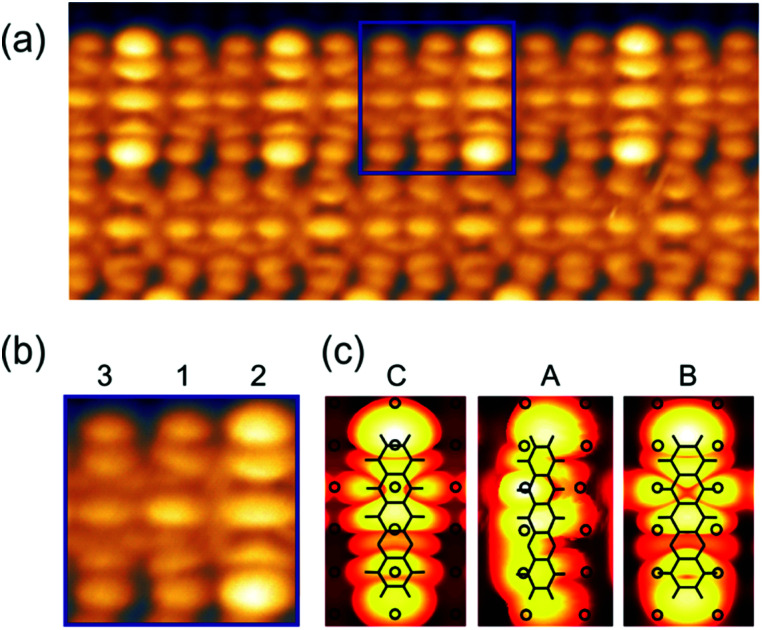
(a) Experimental STM images of DHTAP molecules forming chains of the (7 : 9) HOC phase. (b) Zoom into the area highlighted in (a), depicting three neighbouring molecules in different registry with the substrate. (c) Simulated STM images based on the DFT calculations for a single DHTAP molecule in configuration C, A and B in Fig. 2(a), respectively.

**Fig. 4:**
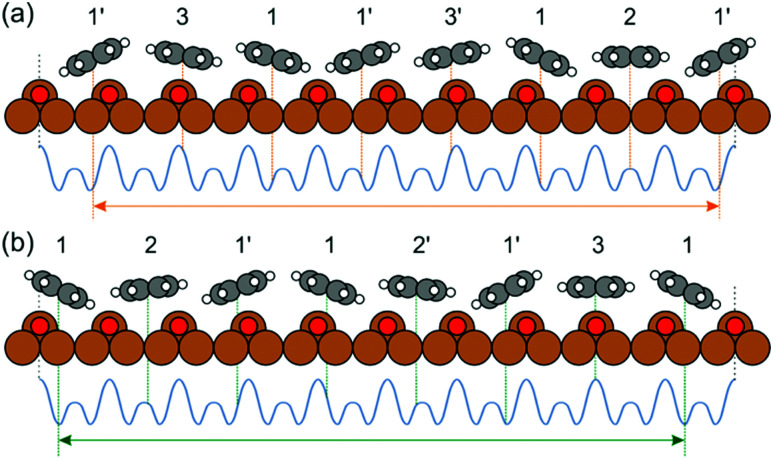
Schematic of the energetically preferred arrangements of the DHTAP molecules in the (7 : 9) HOC phase, assuming a constant separation of the molecules and local configurations (*ϕ*, *z*) interpolated from the DFT results for individual DHTAP molecules. (a) *x*_0_ = 0 and (b) *x*_0_ = *a* (see text).

This additional relaxation energy is counteracted by the loss of the intermolecular binding energy for larger displacements due to the stiffness (force constant *k*) of the molecule interaction potential. However, we expect that *k* could be rather weak since the molecules can compensate changes in the intermolecular spacing by adapting their azimuthal tilt angle. In fact, a similar stress release mechanism has been observed for the adsorption of para-sexiphenyl molecules on the Cu(110)–(2 × 1)O surface, where the flexibility of tilting the molecules stabilizes a (simple) commensurate arrangement along the [1̄10] direction.^18^

We believe that our results are also relevant for other molecular adsorption systems. Indeed, HOC phases have been observed in previous studies.^8–10^ For instance, pentacene on Cu(110)–(2 × 1)O forms a HOC-phase with an epitaxial matrix 
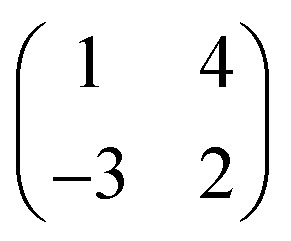
 (see Fig. 1(d and e) in ref. 9) containing three molecules per unit cell. Although the structure is clearly “2D”, a closer inspection reveals that it is actually composed of staggered chains in which the molecules show an alternating image contrast. However, due to the lower quality of the STM image in Fig. 1(d) of ref. 9, it is unclear to what extend the different adsorption sites and/or the molecular conformation (bending, tilt, …) are responsible for the stabilization of this superstructure.

## Conclusions

We have shown that high-order commensurate (HOC) phases can be stabilized through internal degrees of freedoms – in the present case the azimuthal tilt of DHTAP molecules adsorbed on a Cu(110)–(2 × 1)O surface. This stabilization can occur through a combination of several mechanisms: (i) different adsorption configurations of a molecular adsorbate can result in multiple energy minima within the substrate unit cell, thus leading to a strong deviation of the effective corrugation potential from a purely sinusoidal shape. (ii) The configurational flexibility can lead to a significant softening of the intermolecular binding potential and thus facilitate the local relaxation within the HOC unit cell. (iii) Finite domains gain energy if they are terminated by molecules in the energetically preferred adsorption configuration.

## Conflicts of interest

There are no conflicts to declare.

## Supplementary Material
